# Effectiveness of forest honey ( *Apis dorsata*) as therapy for ovarian failure causing malnutrition

**DOI:** 10.12688/f1000research.110660.2

**Published:** 2022-10-20

**Authors:** Erma Safitri, Hery Purnobasuki, Muhammad Thohawi Elziyad Purnama, Shekhar Chhetri

**Affiliations:** 1 Division of Veterinary Reproduction, Department of Veterinary Science, Faculty of Veterinary Medicine, Universitas Airlangga, Surabaya, East Java, 60115, Indonesia; 2 Department of Biology, Faculty of Science and Technology, Universitas Airlangga, Surabaya, East Java, 60115, Indonesia; 3 Division of Veterinary Anatomy, Department of Veterinary Science, Faculty of Veterinary Medicine, Universitas Airlangga, Surabaya, East Java, 60115, Indonesia; 4 Department of Animal Science, College of Natural Resources, Royal University of Bhutan, Lobesa, Punakha, 13001, Bhutan

**Keywords:** forest honey, ovarian failure, malnutrition, oxidative stress, inflammation

## Abstract

**Background: **Malnutrition is the imbalance between intake and nutritional needs, resulting in a decrease in body weight, composition, and physical function. Malnutrition causes infertility due to intestinal and liver degeneration,which may progress to testicular and ovarian degeneration.

**Methods:** An infertile female rat model with a degenerative ovary was induced with malnutrition through a 5-day food fasting but still had drinking water. The administration of (T1) 30% (v/v) and (T2) 50% (v/v) forest honey ( *Apis dorsata*) were performed for ten consecutive days, whereas the (T+) group was fasted and not administered forest honey and the (T−) group has not fasted and not administered forest honey. Superoxide dismutase, malondialdehyde, IL-13 and TNF-α cytokine expressions, and ovarian tissue regeneration were analyzed.

**Results: **Superoxide dismutase was significantly different ( *p*<0.05) in T1 (65.24±7.53), T2 (74.16±12.3), and T− (65.09±6.56) compared with T+ (41.76±8.51). Malondialdehyde was significantly different ( *p*<0.05) in T1 (9.71±1.53), T2 (9.23±0.96), and T− (9.83±1.46) compared with T+ (15.28±1.61). Anti-inflammatory cytokine (IL-13) expression was significantly different ( *p*<0.05) in T1 (5.30±2.31), T2 (9.80±2.53), and T− (0.30±0.48) compared with T+ (2.70±1.57). Pro-inflammatory cytokine (TNF-α) expression was significantly different ( *p*<0.05) in T1 (4.40±3.02), T2 (2.50±1.65), and T− (0.30±0.48) compared with T+ (9.50±1.78). Ovarian tissue regeneration was significantly different ( *p*<0.05) in T− (8.6±0.69) and T2 (5.10±0.99) compared with T1 (0.7±0.95) and T+ (0.3±0.67).

**Conclusion: **The 10-day administration of 50% (v/v) forest honey can be an effective therapy for ovarian failure that caused malnutrition in the female rat model.

## Introduction

Malnutrition in the form of protein–energy malnutrition (PEM) is a challenge in developing countries, including Indonesia. ^1^ Malnutrition is the imbalance between intake and nutritional needs, resulting in a decrease in body weight, composition, and physical function. ^2^ Furthermore, malnutrition contributes to approximately 45% of deaths among children under 5 years old. ^3^ PEM accompanied by diarrhea has been reported to contribute >50% of deaths among children. ^4^ In experimental animals, PEM causes infertility due to intestinal ^5^ and liver degeneration, ^6^ which may progress to testicular ^7^^–^^9^ and ovarian degeneration. ^10^

Malnutrition is closely related to oxidative stress, which is an increase in reactive oxygen species (ROS) that causes damage to cellular components, such as DNA, proteins, and lipids. ^11^ The binding between ROS and lipids can lead to increased levels of malondialdehyde (MDA), a biomarker of increased lipid peroxidation. ^12^ The increased ROS in malnutrition conditions can cause a decrease in the amount of antioxidants in the body. One of the antioxidants that play a significant role in protection from ROS reactions is superoxide dismutase (SOD). ^13^ SOD is an essential enzyme (scavenger) that plays a role in preventing the oxidation process. Decreased antioxidant protection, such as SOD, can lead to various disorders in the form of an immunological response, such as an excessive inflammatory process.

Inflammation is one of the responses of the body’s immune system in recognizing and eliminating harmful components, thereby promoting the healing process. The inflammatory process involves communication between various components in the body. Several components involved in the inflammatory process include tumor necrosis factor alpha (TNF-α) and interleukin 13 (IL-13). TNF-α and IL-13 are cytokines that are formed in response to inflammatory reactions. The two cytokines act antagonistically. TNF-α is a pro-inflammatory cytokine that plays a role in systemic inflammation and one of the cytokines that complete the acute phase reaction, ^14^ whereas IL-13 is an anti-inflammatory cytokine. TNF-α is primarily produced by activated macrophages although can be produced by other cells. The anti-inflammatory response is controlled mainly by IL-13, which is a multifunctional cytokine. ^15^

According to several previous studies, PEM can be overcome by honey administration. ^7^^–^^9^^,^^16^ Honey has various benefits both as a food source and for medicinal purposes, including antibacterial, anti-inflammatory, anti-apoptotic, and antioxidant properties. ^17^^–^^19^ Honey consists of various compounds, which are divided into major and minor compounds. The major compounds are carbohydrates in the form of monosaccharides (fructose and glucose), disaccharides (sucrose, maltose), and oligosaccharides; whereas the minor compounds are amino acids, enzymes, vitamins, minerals, and polyphenols. ^17^^,^^20^ Honey is grouped into two types: monofloral (derived from one type of flower) and polyfloral (more than one type of flower). ^21^ The honey sample in which one pollen type is predominant (>45%) is called unifloral/monofloral. In contrast, absence of predominant pollen type within a honey sample is treated as multiflral/polyfloral. ^18^^,^^19^

Forest honey from *Apis dorsata* bees is one example of polyfloral honey that can be found in Indonesia, where bees get nectar from several types of plants found in a certain area where the forest is located. ^22^ The phenolic and flavonoid content of forest honey ( *A. dorsata*) is a strong combination as an antioxidant. ^23^ The antioxidants possessed by forest honey ( *A. dorsata*) have a higher value than those of monofloral honey. ^19^

Some studies have been performed regarding the administration of honey. ^5^^,^^16^^,^^24^^,^^25^ Homing and differentiation of stem cells were expected in the honey administration in the animal model with ovarian failure. ^5^ Stem cells are derived and differentiated by culture originating from the body itself, facilitating follicle regeneration in the ovary. Ovarian regeneration can be proven by molecular and microscopic studies. ^26^^,^^27^ The microscopic histological appearance will reveal ovarian tissue regeneration at the molecular level, wherein several expressions, such as cluster of differentiation like CD45+ and CD34+ from biomarker of hematopoietic stem cells, ^5^^,^^28^ expression of transforming growth factor-β, ^24^ growth differentiation factor-9, ^26^^,^^27^ vascular endothelial growth factor, and granulocyte colony-stimulating factor of the ovary, were evident. ^24^^,^^29^^,^^30^

Honey has properties that promote wound healing from several antibacterial agents, stimulate the growth of wound tissue, and facilitate an anti-inflammatory response, which rapidly reduces pain, edema, and exudate production. ^31^ Therefore, it is necessary to know about the effect of forest honey ( *A. dorsata*) on SOD and MDA levels, TNF-α and IL-13 expressions, and ovarian tissue regeneration in female albino rats ( *Rattus norvegicus*) experiencing PEM.

## Methods

### Ethical approval

This study was approved by the ethical committee through the Ethical Clearance institution (Komisi Etik Penelitian), Animal Care and Use Committee, Faculty of Veterinary Medicine, University of Airlangga, Surabaya, Indonesia (Number 065-KE).

### Ovarian tissue degeneration of female rats

Ovarian tissue degeneration was achieved by performing a study using a female rat model. Very healthy female Wistar rats ( *R. norvegicus*) with a body weight of 250–300 g each, 8–10 weeks old, were used in this case study. The female rats went without food for 5 days, although they were provided with water. ^5^^,^^10^ The rats were placed in individual plastic cages in the Experimental Animal Laboratory at the Veterinary Medicine Faculty, Universitas Airlangga. Experimental animal laboratories were designed with adequate air circulation, humidity and temperature regulation. In addition, the use of litter and counterflow replacement was performed to ensure eligibility during the study.

### The administration of honey on the malnutrition-induced animal model

A total of 40 rats were divided into four groups as follows: normal rats, without honey (T−); infertile rats, without honey (T+); infertile rats administered 30% (v/v) honey for 10 days (T1); and infertile rats administered 50% (v/v) honey for 10 days (T2).

Forest honey ( *A. dorsata*) from the forest in Batu Malang East Java, Indonesia, was used in this study. MDA and SOD levels, TNF-α and IL-13 expressions, and subsequent folliculogenesis and ovarian tissue regeneration were analyzed. The analysis of MDA and SOD levels was performed using the ELISA method. ^32^^,^^33^ Pro-inflammatory and anti-inflammatory properties of TNF-α ^14^ and IL-13 ^15^ expressions were analyzed using the immunohistochemical (IHC) method in the ovarian tissue. ^5^^,^^10^ Folliculogenesis was indicated by an increase in the follicle De Graaf expression ^34^ and ovarian tissue regeneration using routine hematoxylin and eosin (H&E) staining. ^10^

### MDA and SOD level analysis in serum

The analysis of MDA and SOD levels in serum was performed using the double-antibody sandwich ELISA kit. ^32^^,^^33^ The working principle of this kit is identified by precoated capture antibody (anti-rat MDA monoclonal antibody/anti-rat SOD monoclonal antibody) and detection antibody (biotinylated polyclonal antibody) simultaneously. Furthermore, staining was performed using a substrate of 3,3′,5,5′-tetramethylbenzidine (TMB). TMB reacts through peroxidase activity to form a blue color, and the addition of a stop solution causes a yellow color change. Color intensity has a positive correlation with the target analyte quantity being analyzed.

Serum sample preparation was performed by cooling the extracted blood at 4°C for one night. The serum from the blood sample that has been coagulated and contained in the top layer was then separated and centrifuged for 10 min at a speed of 1,000–3,000 rpm. The supernatant formed can be directly used in ELISA testing or stored (lasts for 1–3 months if stored at a temperature of −20°C to −80°C).

The ELISA test was performed by preparing wells from the ELISA plate of serum samples, standards, and blanks. Initially, 100 μL of serum and blank samples were added to each well and incubated at 37°C for 90 min, and the ELISA plate was subsequently washed two times using a 350-μL wash buffer in each well. After, the liquid was removed by placing the blotting paper on the ELISA plate to remove the liquid. Then, a 100-μL biotinylated polyclonal antibody was added to each well and incubated at 37°C for 30 min. The ELISA plate was then washed five times and dried using the abovementioned method. Next, 100 μL of TMB was added to each well and incubated at 37°C until a color gradient was formed with a maximum time of 30 min. Then, 100 μL of stop solution was added, and the ELISA plate was subsequently read at 450 nm optical density. ELISA plate readings were immediately performed.

### IHC methods for TNF-α and IL-13 analyses

IHC analysis was performed to determine the expressions of TNF-α ^14^ and IL-13. ^15^ First, an incision was made through the ovarian tissues transversely from paraffin blocks. IHC techniques were performed using monoclonal antibodies anti-TNF-α and IL-13. TNF-α and IL-13 expression analyses were performed using a light microscope with a magnification of 400×. TNF-α and IL-13 expressions were indicated by the number of cells with brownish discoloration due to DAB-chromogen in each incision. ^35^ The five fields of view were assessed for each slide through a scoring system. The following IHC scoring system was used: IHC score=A×B, wherein A denotes the wide percentage of expressions and B is the intensity of the chromogen color ( Table 1). ^36^

**Table 1.** Semiquantitative IHC scale taking into account both percentage of positive cells (A) and intensity of reaction color (B) with the final score representing the product of the two variables (A **×** B). ^36^

A	B
0 points no cells with positive reaction	0 points no color reaction
1 point to 10% cells with positive reaction	1 point low intensity of color reaction
2 points 11%−50% cells with positive reaction	2 points moderate intensity of color reaction
3 points 51%−80% cells with positive reaction	3 points intense color reaction
4 points >80% cells with positive reaction	

### Histological and follicle De Graaf analyses of the ovary

The identification of follicle De Graaf and ovarian tissue regeneration was performed using light microscopy examination. ^34^ Histological preparations were performed, including fixing the rat ovary in 10% buffer formalin; dehydrating using a series of alcohol, that is, 70%, 80%, 90%, and 96% (absolute); and clearing of the rat ovary in xylene solution. The tissues were infiltrated with liquid paraffin, which is an embedding agent. Sectioning was performed with a microtome that could be set with a distance of 4–6 μm, and the sections were placed on a slide. The embedding process must be reversed to get the paraffin wax out of the tissue and allow water-soluble dyes to penetrate the sections. Therefore, before any staining can be performed, the slides are “deparaffinized” by running them through xylenes to alcohols to water. Routine H&E staining was used. The stained section was subsequently mounted with Canada balsam, and a coverslip was placed. Analyses and identifications of follicle De Graaf and ovarian regenerations are based on the histological measures of the normal tissue. ^5^

### Statistical analysis

The MDA concentration and SOD activity, TNF-α and IL-13 expressions, and growing follicle count were statistically analyzed using SPSS 15 (SCR_016479) for Windows XP with the level of significance set at 0.05 ( *p*=0.05) and the confidence level at 99% (α=0.01). Steps of comparative hypothesis tests are as follows: test data normality with the Kolmogorov–Smirnov test, homogeneity of variance test, analysis of variance factorial, and *post hoc* test (least significant difference test) using the Tukey HSD 5%.

## Results

The effectiveness of forest honey ( *A. dorsata*) as a therapy for ovarian failure that caused malnutrition was based on the following: increased antioxidant enzyme activity, such as SOD, and decreased oxidative stress concentration, such as MDA; increased anti-inflammatory cytokine expression, such as IL-13, and decreased pro-inflammatory cytokine expression, such as TNF-α; and ovarian tissue regeneration with increased growing follicle count.

The antioxidant activity was analyzed using the ELISA double-antibody sandwich method and was based on increased SOD and decreased MDA concentration as oxidative stress. The SOD analysis showed a significant difference ( *p*<0.05) in T1 (65.24±7.53), T2 (74.16±12.3), and T− (65.09±6.56) compared with T+ (41.76±8.51) ( Table 2). The MDA analysis showed a significant difference ( *p*<0.05) in T1 (9.71±1.53), T2 (9.23±0.96), and T− (9.83±1.46) compared with T+ (15.28±1.61) ( Table 2).

**Table 2.** The average of MDA concentration, SOD activity, TNF-α and IL-13 score expression, and growing follicle count in the ovarian rat tissue.

Treatments	Average±SD				
	Average MDA concentration (nmol/L)	Average SOD activity (%)	Average score TNF-α expression	Average score IL-13 expression	Average growing follicle count
Fertile female negative control group (T−)	9.83 ^a^±1.46	65.09 ^b^±6.56	0.30 ^a^±0.48	0.30 ^a^±0.48	8.6 ^c^±0.69
Infertile female positive control group (T+)	15.28 ^b^±1.61	41.76 ^a^±8.51	9.50 ^c^±1.78	2.70 ^b^±1.57	0.3 ^a^±0.67
Infertile female with 30% honey v/v group (T1)	9.71 ^a^±1.53	65.24 ^b^±7.53	4.40 ^b^±3.02	5.30 ^c^±2.31	0.7 ^a^±0.95
Infertile female with 50% honey v/v group (T2)	9.23 ^a^±0.96	74.16 ^b^±12.3	2.50 ^ab^±1.65	9.80 ^d^±2.53	5.10 ^b^±0.99

^a–d^Different superscripts in the same column are significantly different ( *p <* 0.005).

**Figure 1.**
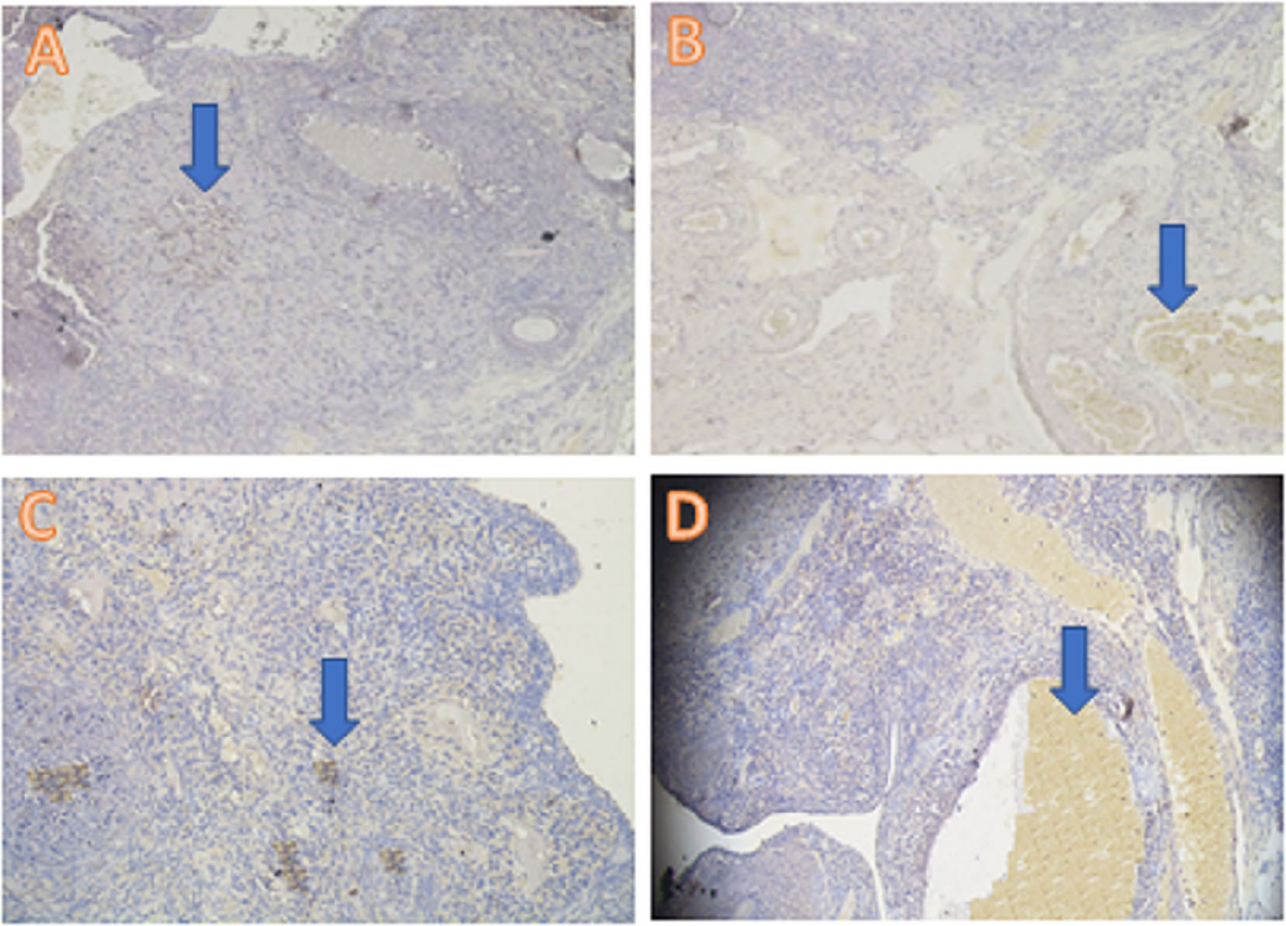
Average score of IL-13 expression (brown): (A) fertile female, negative control group (T−) = 0.30 ^a^ ± 0.48; (B) infertile female, positive control group (T+) = 2.70 ^b^ ± 1.57; (C) infertile female with 30% honey v/v group (T1) = 5.30 ^c^ ± 2.31; (D) infertile female with 50% honey v/v group (T2) = 9.80 ^d^ ± 2.53. (A–D) 400× with the IHC method. IHC = immunohistochemical.

**Figure 2.**
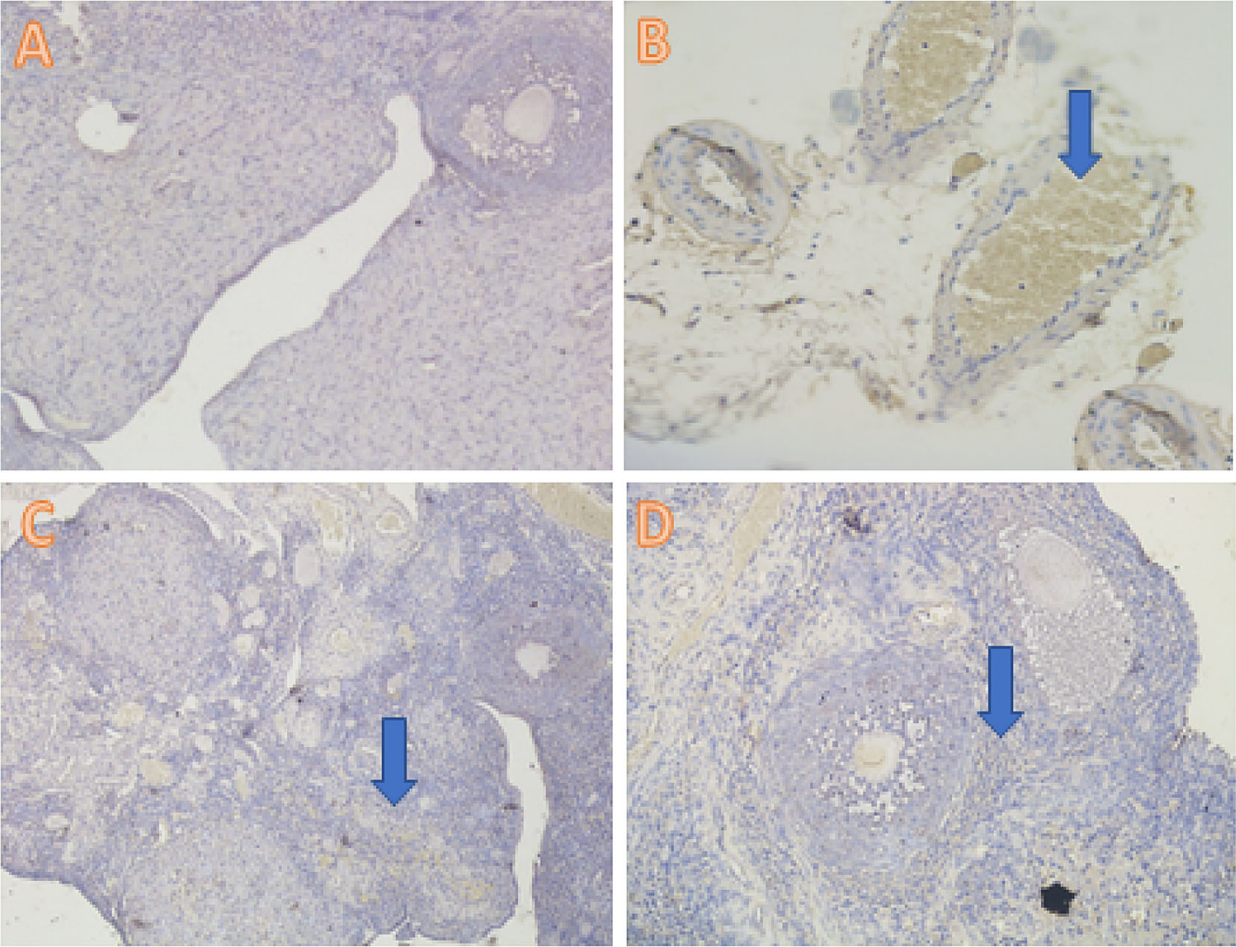
Average score of TNF-α expression (brown): (A) fertile female, negative control group (T−) = 0.30 ^a^ ± 0.48; (B) infertile female, positive control group (T+) = 9.50 ^c^ ± 1.78; (C) infertile female with 30% honey v/v group (T1) = 4.40 ^b^ ± 3.02; (D) infertile female with 50% honey v/v group (T2) = 2.50 ^ab^ ± 1.65. (A–D) 400× with the IHC method. IHC = immunohistochemical.

The anti-inflammatory expression was analyzed using the IHC method and was based on increased IL-13 cytokine expression and decreased TNF-α pro-inflammatory cytokine expression. The IL-3 analysis showed a significant difference ( *p*<0.05) in T1 (5.30±2.31), T2 (9.80±2.53), and T− (0.30±0.48) compared with T+ (2.70±1.57) ( Table 2, Figure 1). The TNF-α analysis showed a significant difference ( *p*<0.05) in T1 (4.40±3.02), T2 (2.50±1.65), and T− (0.30±0.48) compared with T+ (9.50±1.78) ( Table 2, Figure 2).

**Figure 3.**
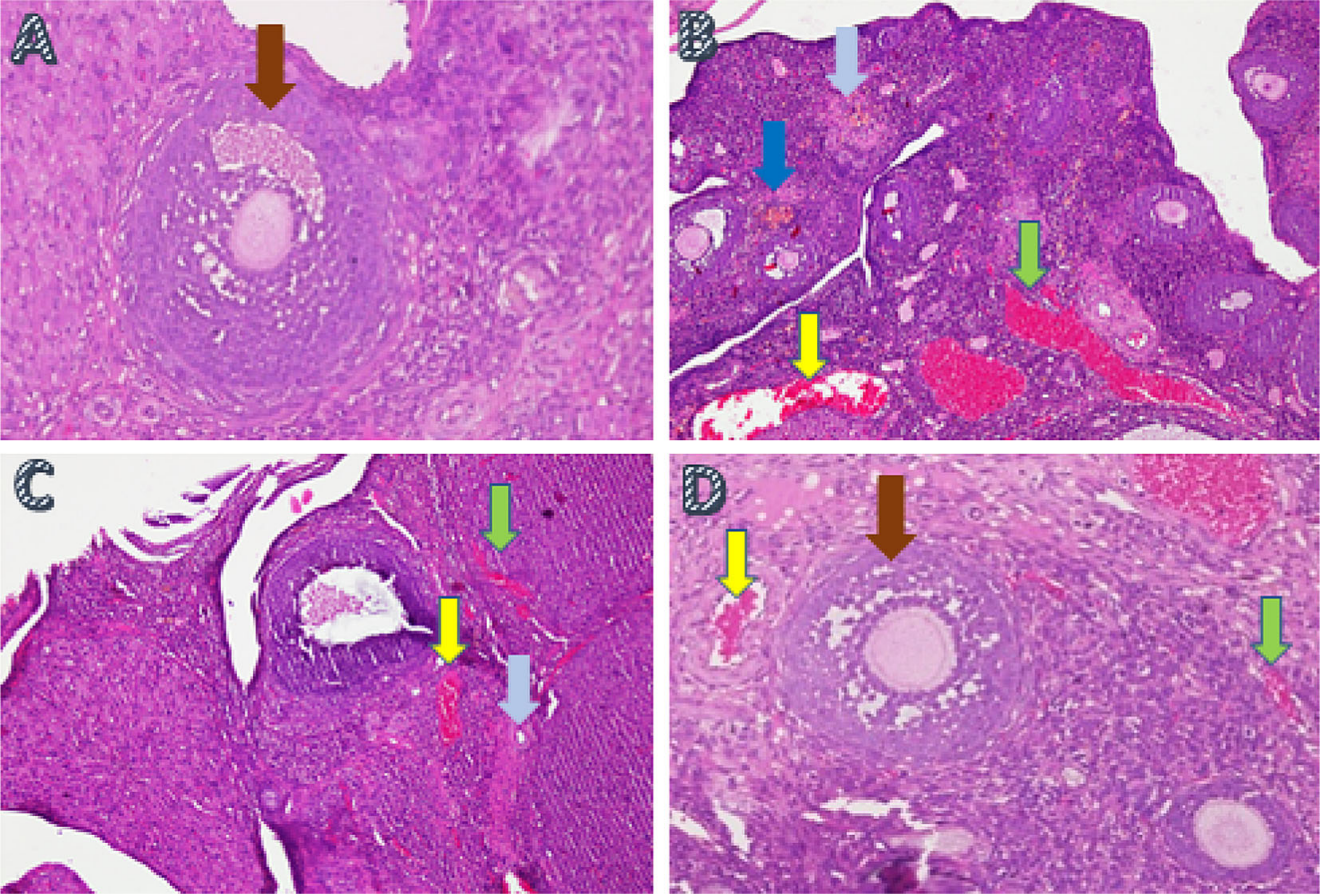
The ovarian tissue regeneration through the method of histopathology anatomy with hematoxylin and eosin (H&E) staining in ovarian rat tissue in a few treatments. A. Fertile female, negative control group (T−): shows growing follicle ( 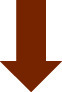); B. Infertile female, positive control group (T+): congestion of the ovary ( 
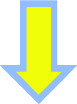
) and widely hemorrhagic ( 
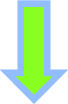
), also visible hemosiderin ( 
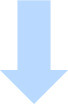
) due to blood cell hemolysis (brownish-yellow color) with fibrin deposition ( 
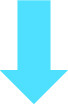
), indicating that chronic congestive has occurred; C. Infertile female with 30% honey v/v group (T1): ovary does not regenerate, congestion appears along the hemosiderin expression and remains widely hemorrhagic; D. Infertile female with 50% honey v/v group (T2): ovaries begin to regenerate, making it appear intact; hemorrhage and congestion still appears in some areas with growing follicles.

Ovarian tissue regeneration was analyzed using the H&E method and was based on the increased growing follicle count. The growing follicle count analysis showed a significant difference ( *p*<0.05) in T− (8.6±0.69) and T2 (5.10±0.99) compared with T1 (0.7±0.95) and T+ (0.3±0.67) ( Table 2, Figure 3).

## Discussion

The increased antioxidant activity and decreased oxidative stress were analyzed using the ELISA double-antibody sandwich method. The increased anti-inflammatory and decreased pro-inflammatory expressions were analyzed using the IHC method, and ovarian tissue regeneration was analyzed using the H&E staining method.

The increased antioxidant activity, such as SOD in T2, can reduce oxidative stress, which allows the MDA concentration to decrease ( Table 2). SOD is a type of essential enzyme that functions as a scavenger against oxidative stress that occurs in the body. Various factors can affect the level and activity of antioxidants in dealing with oxidative stress. Physiological conditions, as well as environmental and genetic conditions, can affect the composition and amount of antioxidants. ^37^ Some researchers say that administering food supplements can also increase the amount of antioxidants in the body. The antioxidants derived from exogenous sources, such as those from food, also have an important role in increasing the endogenous antioxidant activity and neutralizing oxidative stress. ^38^

Decreased antioxidant activity is a sign of oxidative stress conditions. These results are in agreement with the results of another study, which states that nutritional deficiencies can affect the defense system of several scavenger enzymes, such as SOD, glutathione peroxidase, and catalase, in the form of a decreased activity in overcoming oxidative stress. ^39^ Similar results were also found, which stated that antioxidant levels can be significantly decreased ( *p*<0.05) under certain conditions, such as malnutrition. ^40^

Based on the results of this study ( Table 2), significant differences were observed in the T+ group test compared with T1 and T2. The group of infertile rats without forest honey (T+) had a lower activity value and was significantly different ( *p* < 0.05) than the group of infertile rats treated with forest honey (T1 and T2). The significantly higher SOD activity values in the T1 and T2 groups indicated that forest honey therapy could increase the SOD activity in infertile female albino rats due to malnutrition. The results of this study are consistent with those of another study conducted in 2003, wherein the results can prove that the application of natural ingredients of honey can increase the antioxidant activity in the recipient’s blood plasma. ^41^

MDA is a marker of oxidative stress. An increase in MDA indicates an increase in oxidative stress. Malnutrition is one of the causes of oxidative stress. The results of this study are consistent with those of other studies, which state that a lack of nutritional intake can be the cause of oxidative stress, ^7^ which ultimately leads to an increase in MDA concentrations. ^42^

Based on the statistical analysis of the results of this study ( Table 2), a significant difference was noted in the MDA concentration ( *p*<0.05) in T+ compared with T1 and T2. The group of infertile rats without forest honey (T+) had a higher and significantly different MDA concentration than the group of infertile female rats treated with forest honey (T1 and T2), indicating a decrease in oxidative stress conditions in rats administered with forest honey. The results of this study are supported by the results of other studies, stating that honey has an antioxidant property, through a significant decrease in the MDA concentration compared with controls without honey. ^43^^,^^44^

Furthermore, regarding the immune response based on the anti-inflammatory cytokine IL-13 expression, the highest IL-13 expression was found in the T2 treatment group (infertile rats administered with forest honey with a 50% concentration) and the lowest expression was found in the T+ and T− groups. IL-13 is an anti-inflammatory cytokine produced by innate or adaptive immune cells. ^45^ The IL-13 expression that appears indicates that the addition of honey in malnourished rats can reduce the occurrence of inflammatory conditions in the ovarian tissue of experimental albino rats. This is supported by a study conducted in 2016, which states that the administration of honey to malnourished female rats regenerates ovarian tissues. ^5^

Another study in 2016 stated that rats that were not fed for 5 days would experience damage to various organs, including reproductive organs. ^10^ ROS is strongly suspected to be one of the factors that cause organ damage due to malnutrition. Not being fed for a long time and in a row experienced by albino rats as experimental animals in this study can cause an imbalance between the ROS produced and the defense or the presence of antioxidants in the body. This imbalance can ultimately lead to oxidative stress that results in the occurrence of lipid peroxidation in cell membranes, which in turn leads to cell membrane and lipoprotein damage. ^46^

Damage to the cell membrane triggers the release of cellular components that will eventually cause cell death. The emergence of an active immune response occurs as a result of cellular damage. Immune system activation rapidly elicits an acute inflammatory response, which begins with the secretion of various cytokines and chemokines to recruit immune cells to the site of the defect. ^47^

The inflammatory process occurs in response to injury or damage to organs. ^48^ IL-13 is a cytokine that plays a significant role in the anti-inflammatory response. ^15^ In this study, the IL-13 expression appeared in the T1 and T2 treatment groups, wherein the rats received honey therapy. In the negative control group (T−), wherein the condition of the rats was healthy, it could be inferred that IL-13 was not expressed ( Table 2), which was due to the absence of injury in healthy rats. However, in the positive group (T+), wherein the rats were injured and without forest honey, the IL-13 expression was also low.

Forest honey has the highest antioxidant content than other types of honey; therefore, it has an optimal effect on wound healing and inflammation. ^49^ Phenolic compounds are contained in honey and are factors that have a major influence on antioxidant and anti-inflammatory activities. ^17^ IL-13 exerts its anti-inflammatory function through the deactivation of monocytes and macrophages and plays a major role in reducing the pro-inflammatory cytokine production. ^50^ Moreover, IL-13 inhibits potentially damaging inflammatory responses and plays a role in blocking antigen presentation by dendritic cells as well as blocking the activation and infiltration of macrophages to the site of the defect. ^51^

In this study, the increased IL-13 expression proves that forest honey acts as an anti-inflammatory agent. Another anti-inflammatory activity of honey is the decrease in the production of pro-inflammatory cytokines or inflammatory transcription factors, such as NF-κB and MAPK. ^52^ The increased IL-13 expression indicates that the body’s response, through the addition of forest honey, toward tissue damage can be improved. The increase in IL-13 expression in the T1 and T2 forest honey therapy groups showed that the inhibitory reaction to inflammation that occurred was also influenced by the presence of honey therapy.

The next observation is the effectiveness of honey therapy based on a decrease in pro-inflammatory cytokines. Based on the results of this study, the lowest TNF-α expression was in the T2 group, which received the highest forest honey therapy (50% v/v), whereas the highest TNF-α expression was found in the infertile rat group without honey (T+). This indicates that the greatest inflammatory reaction occurred in the malnourished condition in the positive control group (T+) rats. TNF-α is an inflammatory cytokine produced by macrophages or monocytes during acute inflammatory events. TNF-α further contributes to a wide range of cell signaling, causing cell death, such as necrosis or apoptosis. ^53^ TNF-α is mainly secreted by macrophages to stimulate the induction of systemic inflammation. ^54^

Prolonged starvation conditions that cause malnutrition in rats cause damage to various organs, including the ovaries, due to an imbalance between ROS production and the rat body’s antioxidant defenses. In a study conducted in 2016, it was stated that there was severe damage to cells from the ovarian tissue of rats that were not fed for five consecutive days. ^10^ Excessive amounts of ROS in cells can cause damage to cell components, including cell membranes, lipids, proteins, nucleic acids, and other organelles. ^55^ ROS at high concentrations is damaging to cells because ROS can oxidize proteins and lipid cellular components and injure DNA in the cell nucleus. ^47^ The body responds to damage or defects in tissues with the appearance of an inflammatory reaction. ^48^ Inflammation itself is an important part of innate immunity and is regulated by several mechanisms, one of which is through the cytokine mechanism. One of the cytokines that play an important role in the inflammatory response is TNF-α. TNF-α is a pro-inflammatory cytokine that is rapidly released during trauma or infection and is an early mediator in inflamed tissues. ^56^ Inflammation has the aim of eliminating irritant agents and accelerating tissue regeneration. TNF-α signals through two membrane receptors, namely TNFR1 and TNFR2. ^57^ Signaling via TNFR1 and TNFR2 that activates NF-κB and MAPK induces inflammation, tissue regeneration, cell survival, and proliferation, and regulates immune defense against pathogens. ^58^ TNF-α increases the synthesis of anti-inflammatory factors, such as IL-13, corticosteroids, or prostanoids, which can regulate TNF-α expression. ^57^ That if anti-inflammatory factors cannot balance TNF-α, excessive inflammation occurs. In this study, the decrease in TNF-α expression observed in rats administered with forest honey, both at concentrations of 30% v/v and 50% v/v showed that the decrease in inflammatory reactions that occurred was also influenced by forest honey therapy.

In this study, ovarian tissue regeneration, which is shown as an intact ovarian tissue with growing follicles, is the third determinant of the effectiveness of forest honey administration. Ovarian regeneration can be observed microscopically using H&E staining. ^59^^,^^60^ Microscopic examination showed that 50% v/v forest honey therapy (T2), which leads to ovarian tissue repair. Improvements are identified based on the regeneration of the ovary with growing follicles. Overview of these improvements can be compared with the negative control group (T−), which did not suffer from ovarian failure and remained in normal condition with growing follicles ( Figure 3). The abnormal feature of the damaged ovary can be compared with the positive control group of rats (T+) with ovarian failure (degenerative). The microscopic examination showed congested, and severe hemosiderosis (yellow-brown color) was observed owing to the hemolysis of red blood cells with fibrin deposition and then hemorrhage, indicating that chronic congestion has occurred ( Figure 3).

## Conclusions

Honey has properties to stimulate wound healing and facilitate an anti-inflammatory response. It is necessary to know about the effect of forest honey ( *A. dorsata*) on SOD and MDA levels, TNF-α and IL-13 expressions, and ovarian tissue regeneration in female albino rats with PEM condition. Therapy of 50% v/v forest honey for ten consecutive days in female rats with ovarian failure reveals the following findings: increased antioxidant enzyme activity, such as SOD, and decreased oxidative stress concentration, such as MDA; increased anti-inflammatory cytokine expression, such as IL-13, and decreased pro-inflammatory cytokine expression, such as TNF-α; and ovarian tissue regeneration with increased growing follicle count.

## Data availability

### Underlying data

Figshare: Raw data of growing follicle, MDA concentration, TNF-alfa, IL-13 and SOD activity.

https://doi.org/10.6084/m9.figshare.19173857.v3. ^61^

This project contains the following underlying data:

• anova_growing follicle.xlsx
• anova_MDA concentration.xlsx
• anova_TNF-alfa.xlsx
• anova_IL-13 expression.xlsx
• anova_SOD Activity.xlsx

Figshare: Immunohistochemical reaction figures on TNF-alpha.

https://doi.org/10.6084/m9.figshare.19397636.v2. ^62^

This project contains the following underlying data:

• Fig. 1 TNF A.jpeg
• Fig. 2 TNF A.jpeg
• Fig. 3 TNF A.jpeg
• Fig. 4 TNF A.jpeg

Figshare: Histopathological figure: Ovary.

https://doi.org/10.6084/m9.figshare.19397630.v2. ^63^

This project contains the following underlying data:

• Fig. 1 HE.jpeg
• Fig. 2 HE.jpeg
• Fig. 3 HE.jpeg
• Fig. 4 HE.jpeg

## Reporting guidelines

Figshare: ARRIVE checklist for ‘Effectiveness of forest honey ( *Apis dorsata*) as therapy for ovarian failure that caused malnutrition’, https://doi.org/10.6084/m9.figshare.19642266.v1. ^64^

Data are available under the terms of the Creative Commons Attribution 4.0 International license (CC-BY 4.0).
