# Supplementary material for: Effectiveness of forest honey ( Apis dorsata) as therapy for ovarian failure causing malnutrition
Source: F1000Res. 2022 Oct 20;11:512. Originally published 2022 May 12. [Version 2] doi: 10.12688/f1000research.110660.2 (PMC10521050; doi:10.12688/f1000research.110660.2)
Supplement: Peer review history

# Reviewer report: Reviewer response for version 2

**DOI:** 10.5256/f1000research.139655.r153735

**Published:** 2022-10-21

**Authors:** Ujjwal Layek

**Affiliations:** 1 Department of Botany, Rampurhat College, Rampurhat, West Bengal, India

The authors are well responded to the comments. I think the manuscript will be suitable for indexing after the following revisions:

1. The honey sample in which one pollen type .......... treated as multiflral/polyfloral. ----- here, "multiflral" will be "multifloral"
2. Forest honey from Apis dorsata bees is one example of polyfloral honey........ I think adding author citation to the scientific name for the first time within the text will be better. " *Apis dorsata*" to " *Apis dorsata* Fabricius"
  Authors may also rewrite the sentence as - Forest honey from giant honeybees ( *Apis dorsata* Fabricius) is one example ...... 

Is the work clearly and accurately presented and does it cite the current literature?

Partly

If applicable, is the statistical analysis and its interpretation appropriate?

Yes

Are all the source data underlying the results available to ensure full reproducibility?

Yes

Is the study design appropriate and is the work technically sound?

Partly

Are the conclusions drawn adequately supported by the results?

Yes

Are sufficient details of methods and analysis provided to allow replication by others?

Yes

Reviewer Expertise:

Plant-pollinator interactions, Honeybee biology, stingless bee biology

I confirm that I have read this submission and believe that I have an appropriate level of expertise to confirm that it is of an acceptable scientific standard.

# Reviewer report: Reviewer response for version 1

**DOI:** 10.5256/f1000research.122287.r143797

**Published:** 2022-09-15

**Authors:** Aulanni’am Aulanni'am, Husnul Khotimah

**Affiliations:** 1 Department of Biochemistry, Faculty of Mathematics and Natural Sciences, Universitas Brawijaya, Malang, Indonesia; 2 Department of Pharmacology, Faculty of Medicine, Universitas Brawijaya, Malang, Indonesia

The Title: will be better if showed the object (Rat)

 The dose of Honey, will be stronger for conclusion if you have 3 doses, so we will know the trends.

 This is good research. however, I had some questions :

1. In this study you used IHC (immunohistochemistry) for measuring TNFa as an inflammatory mediator rather than ELISA because by ELISA we will know the level of TNFa directly.
2. This study also only used 2 doses of forest honey. because pharmacologically at least 3 doses are needed to know the trends and dose-response curve.
3. There is such a jumping discussion about tissue damage. I thought tissue damage was the end point of this research, and several variables (MDA, SOD, TNFa and IL-13) is an intermediate variables that explained the endpoint.

Overall, this is a good experiment with good results

Is the work clearly and accurately presented and does it cite the current literature?

Yes

If applicable, is the statistical analysis and its interpretation appropriate?

Yes

Are all the source data underlying the results available to ensure full reproducibility?

Yes

Is the study design appropriate and is the work technically sound?

Yes

Are the conclusions drawn adequately supported by the results?

Yes

Are sufficient details of methods and analysis provided to allow replication by others?

Yes

Reviewer Expertise:

Molecular Biochemistry: the molecular mechanism of carbohydrate, protein and lipid metabolism;  molecular pharmacology: mechanism or drugs, herbal or natural resources; animal model: a design for some animal models such as diabetes, cancers, infertility, malnutrition

We confirm that we have read this submission and believe that we have an appropriate level of expertise to confirm that it is of an acceptable scientific standard.

# Response

**Published:** 2022-10-09

**Authors:** Muhammad Thohawi Elziyad Purnama

**Affiliations:** Airlangga University, Indonesia

Dear Reviewer, 

 Thanks for your valuable comments and suggestions on the manuscript entitled:

 Effectiveness of forest honey (Apis dorsata) as therapy for ovarian failure causing malnutrition

 We welcome feedback. We have made modifications to the manuscript according the track comment.

# Reviewer report: Reviewer response for version 1

**DOI:** 10.5256/f1000research.122287.r149430

**Published:** 2022-09-15

**Authors:** Ujjwal Layek

**Affiliations:** 1 Department of Botany, Rampurhat College, Rampurhat, West Bengal, India

The authors determine the effect of honey feeding on the fertility of rats. They measured superoxide dismutase (SOD) and malondialdehyde (MDA) levels and observed ovarian tissue regeneration in different treatments. I think the work has a significant impact on medical science. However, the manuscript needs to improve by focusing on the following: 

 Abstract

- Background: needs to focus on the effect of malnutrition on fertility.
- Results: SOD, MDA; I think full forms will be better for readers.

Introduction

- Inflammation due to malnutrition is needs to be illustrate.
- "monofloral" delete it.
- "medical purposes ......... antioxidant properties".---- use more references.
- "Monofloral (derived ...) and polyfloral.... )." --- The honey sample in which one pollen type is predominant (>45%) is called unifloral/monofloral. In contrast, absence of predominant pollen type within a honey sample is treated as multiflral/polyfloral.
  - Vonder Ohe et al. 2004. Apidologie 35: S18-S25. doi: 10.1051/apido:2004050
  - Layek and Karmakar 2018. Grana 57(4): 298-310. doi: 10.1080/00173134.2017.1390604
- Apis dorsata ----- add author citation Fabricius
- "Forest honey ...... in Indonesia." --- why polyfloral? Monofloral and polyfloral depend on surrounding vegetation. Mention plants and add references.

Methods

- MDA and SOD ....
  - 100-uL---- correct

Figure ligands: need to be concise

 Conclusion

- add text about the importance of the work

References

- several errors are there and do not maintain the journal's style

Is the work clearly and accurately presented and does it cite the current literature?

Partly

If applicable, is the statistical analysis and its interpretation appropriate?

Yes

Are all the source data underlying the results available to ensure full reproducibility?

Yes

Is the study design appropriate and is the work technically sound?

Partly

Are the conclusions drawn adequately supported by the results?

Yes

Are sufficient details of methods and analysis provided to allow replication by others?

Yes

Reviewer Expertise:

Plant-pollinator interactions, Honeybee biology, stingless bee biology

I confirm that I have read this submission and believe that I have an appropriate level of expertise to confirm that it is of an acceptable scientific standard, however I have significant reservations, as outlined above.

## References

[1] : Harmonized methods of melissopalynology. *Apidologie* .2004;35(Suppl. 1) : 10.1051/apido:2004050 S18-S25 10.1051/apido:2004050

[2] : Pollen analysis ofApis dorsata Fabricius honeys in Bankura and Paschim Medinipur districts, West Bengal. *Grana* .2018;57(4) : 10.1080/00173134.2017.1390604 298-310 10.1080/00173134.2017.1390604

# Response

**Published:** 2022-10-09

**Authors:** Muhammad Thohawi Elziyad Purnama

**Affiliations:** Airlangga University, Indonesia

Dear Reviewer, 

 Thanks for your valuable comments and suggestions on the manuscript entitled:

 Effectiveness of forest honey (Apis dorsata) as therapy for ovarian failure causing malnutrition

 We welcome feedback. We have made modifications to the manuscript according to the following:

 Abstract

 Background: needs to focus on the effect of malnutrition on fertility.

**Answer: **We have revised with the focus research contribution.

 Results: SOD, MDA; I think full forms will be better for readers.

**Answer: **We have revised with the full form of SOD and MDA.

 Introduction

 Inflammation due to malnutrition is needs to be illustrate.

**Answer: **We have revised.

 "monofloral" delete it.

**Answer: **We have revised.

 "medical purposes ......... antioxidant properties".---- use more references.

**Answer: **We have added a reference.

 "Monofloral (derived ...) and polyfloral.... )." --- The honey sample in which one pollen type is predominant (>45%) is called unifloral/monofloral. In contrast, absence of predominant pollen type within a honey sample is treated as multiflral/polyfloral.

 Vonder Ohe et al. 2004. Apidologie 35: S18-S25. doi: 10.1051/apido:2004050

 Layek and Karmakar 2018. Grana 57(4): 298-310. doi: 10.1080/00173134.2017.1390604

 Answer: We have added this two reference.

 Apis dorsata ----- add author citation Fabricius

**Answer:** We have revised.

 "Forest honey ...... in Indonesia." --- why polyfloral? Monofloral and polyfloral depend on surrounding vegetation. Mention plants and add references.

**Answer: **We have revised.

 Methods

 MDA and SOD ....

 100-uL---- correct

**Answer:** We have revised.

 Figure ligands: need to be concise

**Answer:** We have revised.

 Conclusion

 add text about the importance of the work

**Answer: **We have added the brief conclusion regarding these finding.

 References

 several errors are there and do not maintain the journal's style

**Answer: **We have revised all references according to journal's style.
